# Practical Implementation of Artificial Intelligence-Based Deep Learning and Cloud Computing on the Application of Traditional Medicine and Western Medicine in the Diagnosis and Treatment of Rheumatoid Arthritis

**DOI:** 10.3389/fphar.2021.765435

**Published:** 2021-12-23

**Authors:** Shaohui Wang, Ya Hou, Xuanhao Li, Xianli Meng, Yi Zhang, Xiaobo Wang

**Affiliations:** ^1^ School of Ethnic Medicine, Chengdu University of Traditional Chinese Medicine, Chengdu, China; ^2^ School of Pharmacy, Chengdu University of Traditional Chinese Medicine, Chengdu, China; ^3^ Chengdu Second People’s Hospital, Chengdu, China; ^4^ State Key Laboratory of Southwestern Chinese Medicine Resources, Innovative Institute of Chinese Medicine and Pharmacy, Chengdu University of Traditional Chinese Medicine, Chengdu, China

**Keywords:** artificial intelligence, data mining, deep learning, cloud computing, traditional medicine, Tibetan medicine, rheumatoid arthritis

## Abstract

Rheumatoid arthritis (RA), an autoimmune disease of unknown etiology, is a serious threat to the health of middle-aged and elderly people. Although western medicine, traditional medicine such as traditional Chinese medicine, Tibetan medicine and other ethnic medicine have shown certain advantages in the diagnosis and treatment of RA, there are still some practical shortcomings, such as delayed diagnosis, improper treatment scheme and unclear drug mechanism. At present, the applications of artificial intelligence (AI)-based deep learning and cloud computing has aroused wide attention in the medical and health field, especially in screening potential active ingredients, targets and action pathways of single drugs or prescriptions in traditional medicine and optimizing disease diagnosis and treatment models. Integrated information and analysis of RA patients based on AI and medical big data will unquestionably benefit more RA patients worldwide. In this review, we mainly elaborated the application status and prospect of AI-assisted deep learning and cloud computation-oriented western medicine and traditional medicine on the diagnosis and treatment of RA in different stages. It can be predicted that with the help of AI, more pharmacological mechanisms of effective ethnic drugs against RA will be elucidated and more accurate solutions will be provided for the treatment and diagnosis of RA in the future.

## Introduction

Rheumatoid arthritis (RA) is a non-fatal but currently incurable chronic symmetric autoimmune disease that involves multiple bones and joints, and their surrounding tissues, such as wrist joints, knuckles, metacarpophalangeal and metatarsophalangeal joints (Sparks, 2019; Scherer et al., 2020). Meanwhile, it can increase the incidence of venous thromboembolism (Molander et al., 2021), lung diseases (Juge et al., 2018), cardiovascular diseases (Semb et al., 2020), eye diseases (Artifoni et al., 2014), diabetes (Westra et al., 2018; Inamo et al., 2021), periodontal diseases (Courbon et al., 2019) and non-melanoma skin cancer (Assassi, 2016; Raaschou et al., 2016) (Supplementary Figure S1). The epidemiological data show that the incidence of RA is positively correlated with age (the initial episode of RA is commonly of 40–60 years old) (Allen et al., 2018), with the characteristics of genetic tendency (Webster et al., 2018; Okada et al., 2019), regional and ethnic differences (James, 1996; Hughes et al., 2006). Less equally, women are three times more susceptible to RA than men, due to women’s menstrual flow, pregnancy, delivery and breastfeeding behaviors that disrupt sex hormones and deplete Qi and blood (Favalli et al., 2019). At present, the pathogenesis of RA has not been fully clarified and there is no specific radical cure for RA (Croia et al., 2019). Traditional medicine has a long history in the treatment of RA. However, the unknown target and pharmacological mechanism of a single Chinese medicine or preparation hinders the development of traditional medicine. In addition, the irregular diagnosis and treatment regimen of RA leads to uneven therapeutic effects. With the accumulation of big data for RA diagnosis and research, the improvement of computing network, machine learning and other models, AI will bring dawn to the breakthrough of RA diagnosis and treatment as well as drug discovery for RA.

The subtle clinical signs of RA are easier to be confused with several other arthritis diseases (Including osteoarthritis, psoriatic arthritis and gout) at onset. As the disease worsens, severe joint damage, undesirable and outrageous arthralgia and other troublesome complications are extremely distasteful to patients. So, it is pretty desirable to make timely and accurate early diagnosis, reasonable management and prospective prognosis for RA patients. However, there are obvious technical constraints (such as insensitivity, delayed diagnosis of diseases and obvious adverse reactions of biologics) in conventional physician check, joint imaging with diagnostic and treatment instruments, detection of various serum and synovial joint markers in RA patients (Conigliaro et al., 2019; Widdifield, 2019). Although western medicine, as well traditional medicine of various ethnic groups including unique Tibetan medicine bath and other internationally recognized RA therapies has the potential role on RA, its mystery still needs to be uncovered to explore deeper pharmacological mechanisms. Therefore, in addition to conventional approaches, clinicians should also consider the unknown etiology, complex pathologic process, unpredictable prognosis, and vast amounts of ethnic medicine therapy of RA when managing the main and/or suspected symptoms of RA. In this review, we also confirmedly propose to weigh multiple parameters from data mining, AI-based deep learning and digital pathology of RA-related medicine and disease database. And further reasonable introduction of computer algorithms is employed to integrate and analyze seemingly unrelated clinical signs of RA patients. This not only provides a reference for comprehensive management of RA patients, including disease status, optimal treatment and combination strategies, all possible prognostic and elusion tactics for RA. In addition, it is more important to promote the integration of AI and digital medical transformation, realize the integration of traditional medicine and western medicine under the vision of AI, enhance the clinical diagnosis and treatment of RA and drug development, and improve the quality and cost of healthcare.

### Application of AI in Western Medicine and Traditional Medical Healthcare

**FIGURE 1:**
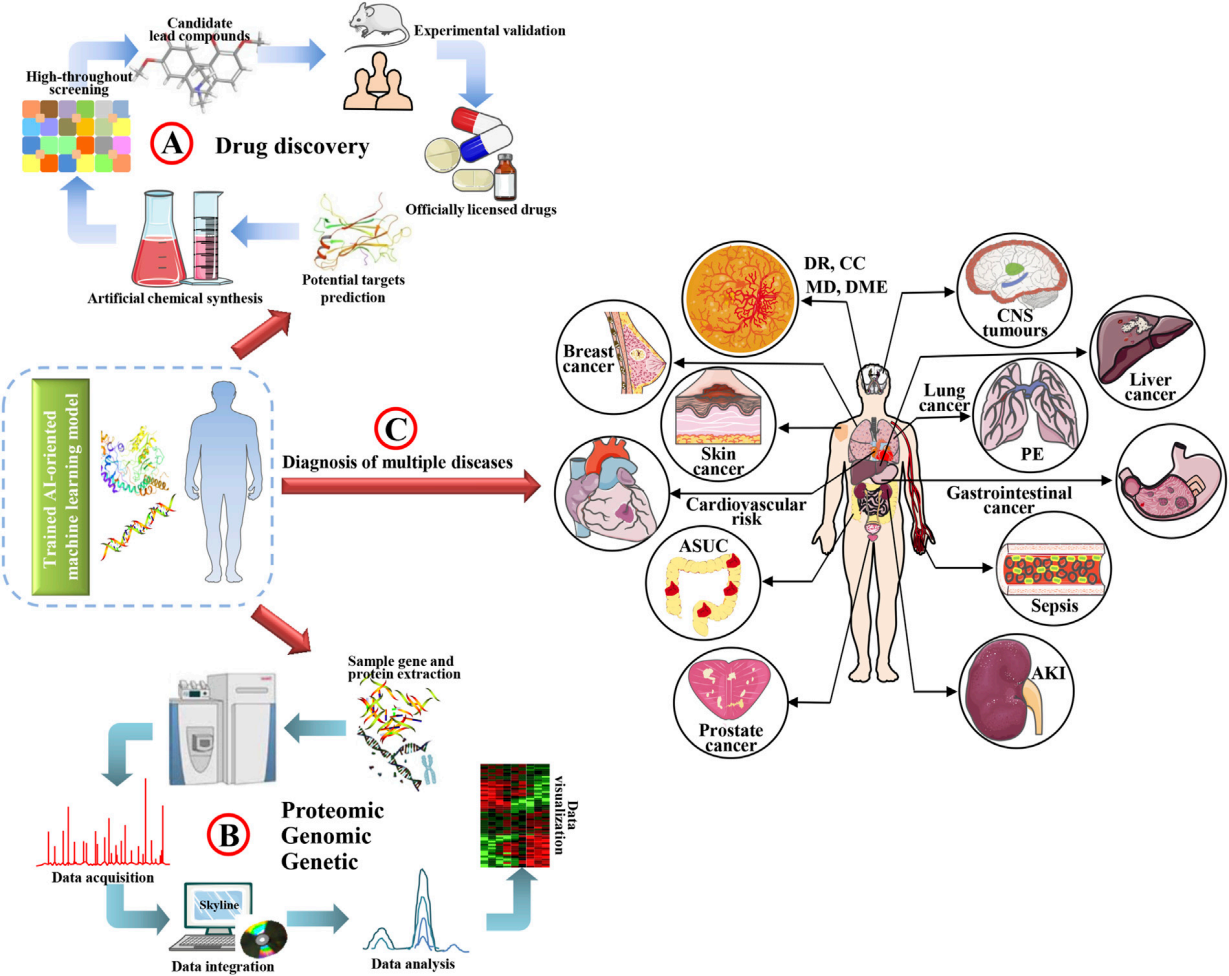
Application framework of AI in medical field.

AI is considered a branch of engineering capable of realizing novel concepts and solutions to solve complex challenges. AI integrated medical big data processing is to give a relatively fast mining of massive existing data such as the cause of intractable diseases, disease-specific clinical examination images, related available drugs, and disease development, outcome and prognosis through multiple open source data analysis platforms, which eventually and accurately diagnose disease and provide individualized reasonable treatment recommendations by comparing clinical signals of patients with simulated artificial algorithms (Torkamani et al., 2017; He et al., 2019; Dzobo et al., 2020). In recent years, clinical and scientific researchers have realized the importance and considerable prospect of AI application in medical fields, especially for complex diseases. The digitalization of medical image acquisition provides convenient conditions for the application of AI. Lee et al. developed a computer-aided diagnosis system based on deep learning for diagnosing cervical lymph node metastasis of thyroid cancer by CT scanning, after eight modes of deep learning were used for detection, the recognition of lymph node metastasis reached 90.4% (Lee et al., 2019a). A trained deep learning algorithm-convolutional neural networks (CNNs) has been developed for detecting diabetic retinopathy in retinal fundus photographs (Wong and Bressler, 2016), and classifying age-related macular degeneration and diabetic macular edema, as well accurately distinguishing bacterial and viral pediatric pneumonia using retinal optical coherence tomography and chest X-rays images, respectively (Kermany et al., 2018). Integrated platform of AI-based deep learning, CNNs and cloud computing has been developed for detection of congenital cataracts (Long et al., 2017). Furthermore, AI has also achieved fine results in the application of pathological diagnosis. Scientists have successfully used machine learning to distinguish fine-grained variability of different types of skin cancer using CNNs (Esteva et al., 2017), DNA methylation-based screening for central nervous system tumors (Capper et al., 2018), and identify clinical phenotypes of sepsis (Seymour et al., 2019). As well constructed an AI learning method that can automatically identify pathological images and clinically inherited non-small cell lung cancer gene mutations, providing a basis for personalized treatment of lung cancer (Kumar et al., 2018). Early diagnosis and treatment of breast cancer are very important to the prognosis of patients, by evaluating and diagnosing breast cancer cell markers, AI can make more patient-appropriate treatment decisions (Robertson et al., 2018). In a word, AI-based deep learning and cloud computing have blossomed in fields such as digital pathological image diagnosis of diseases (Niazi et al., 2019), radiology (Hosny et al., 2018; Van Assen et al., 2020), oncology (Bi et al., 2019), cardiovascular disease (Krittanawong et al., 2019), genetics and genomics (Langmead and Nellore, 2018; Zou et al., 2019), proteomics (Feng et al., 2017; Vishnoi et al., 2020), neurosciences (Perkel, 2018; Choi et al., 2020), and discovery, optimization and verification of drug target and lead compounds in drug discovery (Simm et al., 2018; Raschka and Kaufman, 2020) (Figure 1).

Traditional medicine, especially TCM is a complete medical system with thousands of years of application history, its clinical practice mainly focuses on diagnosis and treatment. TCM has played a crucial role in the treatment of many diseases of Chinese people, such as diabetes, cancer and RA, etc. (Li et al., 2021a; Wang et al., 2021b; Wang et al., 2021d; Xiang et al., 2021). Especially since the outbreak of COVID-19, TCM has played its unique advantages and gradually gained recognition abroad (Hou et al., 2021; Zhao et al., 2021). With the rapid development of science and technology, AI has attracted much attention in the field of TCM. The application of AI has not only observably improved the reliability and accuracy of diagnosis, but also provides favorable conditions for screening suitable TCM or program for disease treatment, as well as promoting the modernization of traditional medicine (Wang et al., 2021d). At present, the establishment of new methods such as systems biology, bioinformatics, integrated pharmacology and network pharmacology has brought new hope for the modernization of TCM research (Ren et al., 2020; Shen et al., 2020; Wang et al., 2021c; Fang et al., 2021). For example, the establishment of databases such as integrative pharmacology-based research platform of traditional Chinese medicine (TCMIP), traditional Chinese medicine database and analysis platform (TCMSP), the encyclopedia of traditional Chinese medicine (ETCM) and other databases based on AI has provided effective tool for revealing potential active ingredients, pharmacological mechanism and compatibility of TCM. Integrated pharmacology based on traditional medicine is regarded as a paradigm shift in TCM (Xu et al., 2021a). Some scholars demonstrated that the five compounds including baicalin, chlorogenic acid, sweroside, phillyrin and forsythoside A were the anti-influenza substances of Shuang-Huang-Lian preparation, and the anti-influenza pharmacological mechanism was related to TNF signaling pathway by serum pharmaco-chemistry and network pharmacology (Zhang et al., 2021a). Other studies have found that the key metabolites of Xihuang Pill enhancing the efficacy of anlotinib for lung cancer management are regulated by multi-component and multi-target interaction networks through comprehensive metabolomics and network pharmacology (Li et al., 2021b). Additionally, we can also use these AI techniques to bridge the relationship between the biological network of disease phenotype and patient genotype as a whole. In this way, we can not only understand the biological basis of TCM diagnosis, but also help us elucidate the mechanism of disease and therapeutic target to establish a new treatment method of targeted biological network therapy combining traditional medicine and western medicine. Such as some scholars used the optimized support vector machine model to establish a diagnosis model of lung cancer based on serological indicators, and a molecular regulation model of Wogonin in *Scutellaria baicalensis*. They finally constructed the regulatory network of Wogonin on cell apoptosis and serological susceptibility genes, which provided a new idea for the clinical diagnosis, treatment and prognosis of lung cancer (Wang et al., 2021a). Zhang et al. proposed a unified intelligent TCM framework based on the edge cloud computing system, which incorporated deep learning algorithm to establish the model for the syndrome differentiation and prescription recommendation through the differentiation of hypertension and cold (Zhang et al., 2021b). The channels and collaterals of TCM are closely related to diseases, which helps to improve the accuracy of clinical medication. Wang et al. used machine learning to predict herbal active ingredients after labeling, manifesting that AI algorithm could explain the relationship between herbal medicines and meridians (Wang et al., 2019b). Moreover, AI technology can be found in the disorderly data of each keyword potential link, and secondary sorting, deep learning and training for many times based on the existing data. By extracting and summarizing from the complex symptoms of TCM, potential association rules between symptoms, prescriptions and core drugs can be determined for clinical diagnosis and treatment (Lu et al., 2021). All in all, with the deepening of the integration of AI and the medical field, the digitalization of the four diagnostic methods of TCM, the intelligent decision system and the modernization of TCM theory will make great progress.

It is not difficult to find that efficient and accurate medical image AI recognition is entering clinical practice. AI can not only facilitate medical diagnostic innovation and assist doctors in making diagnosis and treatment decisions, but also accelerate the discovery of innovative drugs with better efficacy (Xu et al., 2021b). Currently, the application of AI in the medical field is mainly based on a certain module in the process of disease diagnosis and treatment. The establishment of RA auxiliary diagnosis and treatment system requires the combination of the examination and laboratory information of patients, medical record information and joint image data to make decisions, providing recommendations on prescription usage and dosage according to the diagnosis results. In this process, we need to integrate the existing information on the pathogenesis, diagnosis, treatment and patients of RA. Besides, we also need to consider the accuracy of the obtained data and the security of the algorithm, as well as the supplement of new data.

### Understanding of the Nature of RA

#### Current Insight of the Aetiopathogenesis of RA in Western Medicine

**FIGURE 2:**
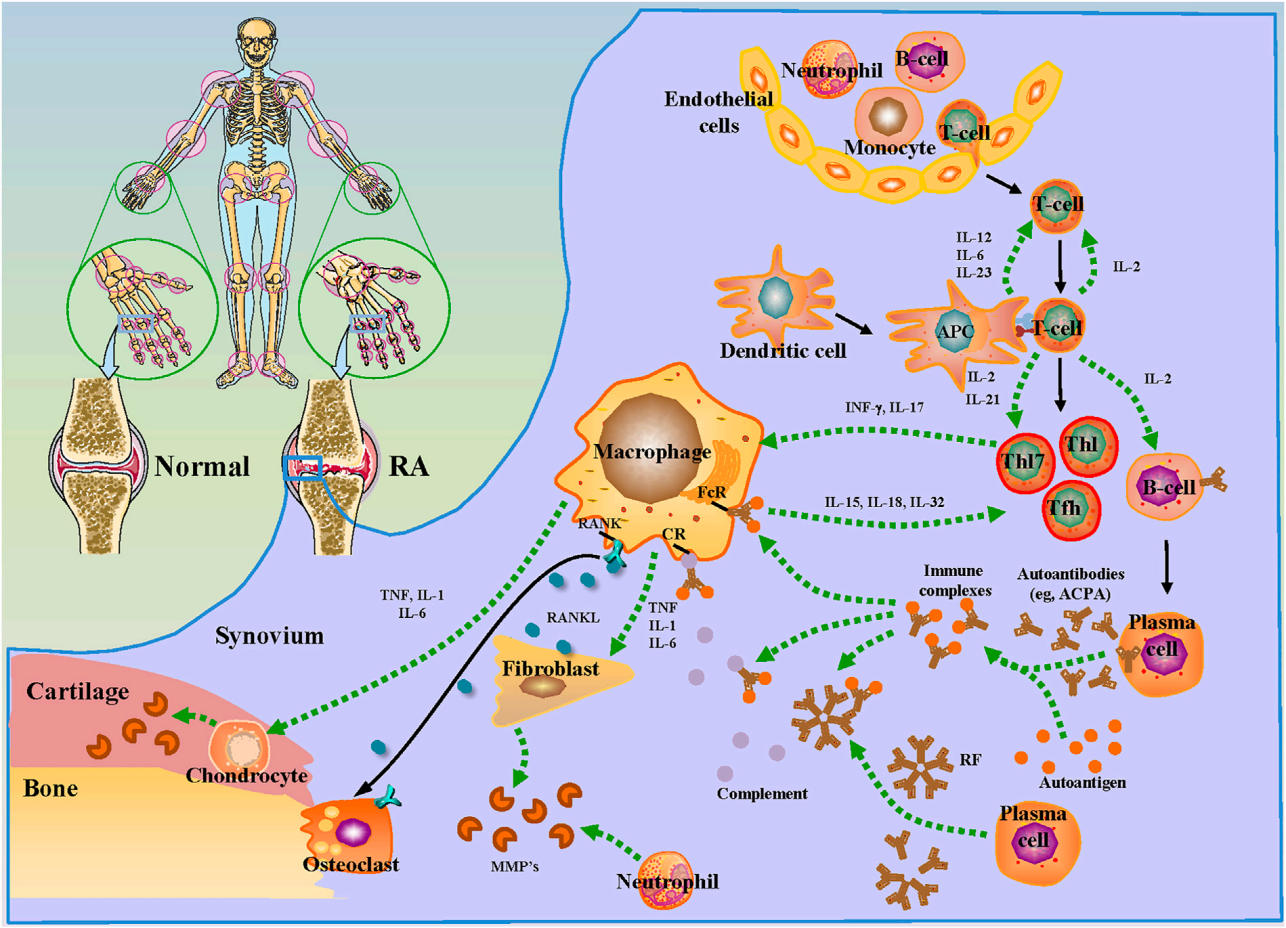
Molecular mechanisms of synovial and cartilage injury in RA patients.

As a chronic autoimmune disease, T cells in the blood of RA patients enter the damaged joints, triggering an inflammatory cascade that causes joint synovium, cartilage, and bone damage (Zhang et al., 2019; Weyand and Goronzy, 2021). The interleukin (IL)-6, 12 and 23 released by dendritic cells stimulated antigen presenting cells to produce IL-2 and 21, contributing to the maturation of T cells. On the one hand, the matured T cells differentiate into helper T (TH)1, TH17 and helper T follicular cells, and release IFN-γ and IL-17 to activate macrophages (Lai Kwan Lam et al., 2008; Rao et al., 2017). Besides, the matured T cells stimulate B cells to differentiate into plasma cells and secrete a large number of autoantibodies such as anti-citrullinated protein antibodies, which formed massive immune complexes of autoantibodies and corresponding antigens in the synovium. After combining with rheumatoid factor (RF), the deposited immune complexes in damaged joints further promote its deterioration (Blüml et al., 2014). Meanwhile, the immune complex and complement binding immune complex bind to Fc and complement receptor of macrophages, respectively. Consequently, the release of tumor necrosis factor (TNF), IL-1 and IL-6 by the activated macrophages stimulate fibroblasts and chondrocytes to release matrix metalloproteinases (MMP) to degrade cartilage (Firestein and Mcinnes, 2017). In turn, it promotes the differentiation of T cells by releasing IL-15, 18 and 32, forming a vicious inflammatory response cycle. In addition to T cells and macrophages, neutrophils and fibroblasts are also involved in the pathological deterioration of RA. Neutrophils can proliferate in the joints of patients with RA and continue to damage the joints by secreting MMP and inducing inflammation. A new study confirms that the reduction in apoptosis and migration caused by the deletion of ELMO1 gene in neutrophils can avoid the aggregation of neutrophils with lower migration ability and alleviate the progressive damage irritated by inflammation to joints (Arandjelovic et al., 2019). Moreover, the receptor activator of nuclear factor kappa B (RANK) released by activated macrophages can combine with fibroblasts-released RANK-ligand (RANKL) to promote the differentiation of osteoclasts and cause bone destruction and cartilage degeneration (Aletaha and Smolen, 2018). Evidence has also shown that fibroblasts located in the synovial lining and sublining can cause bone damage through different mechanisms. Fibroblasts in the inner layer can directly destroy bone and cartilage by secreting FAPα protein, causing bone erosion. While the fibroblasts of synovial sublining secrete a large number of cytokines, triggered by FAPα and Thy-1 proteins, which enhance the immune response of joints (Croft et al., 2019; Wynn, 2019; Chu, 2020) (Figure 2).

**FIGURE 3:**
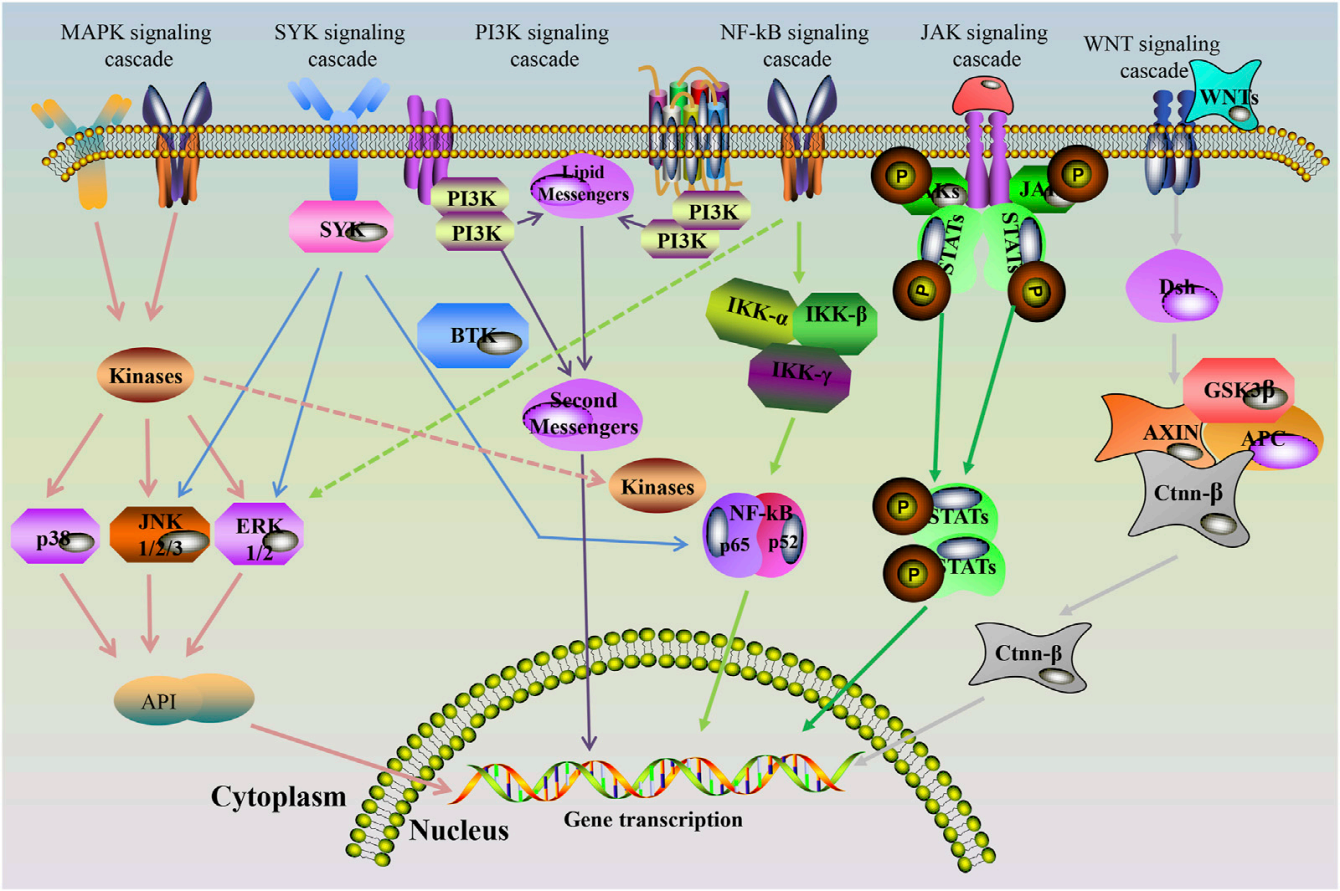
Signaling pathways involved in joint injury in RA patients.

The pathological molecular mechanism of RA is complex and closely related to multiple signal pathways (Catrina et al., 2016; Smolen et al., 2016; Mcinnes and Schett, 2017; Guo et al., 2018; Smolen et al., 2018; Simon et al., 2021) (Figure 3). PI3K/AKT signaling pathway plays an important role in the proliferation and survival of many cells, especially in the regulation of leukocyte migration and fibroblast-evoked cartilage injury. At the same time, TNF-α, IL-17 and other cytokines are stimulated to participate in osteoclast differentiation and generation, and promote osteoclast migration to destroy bone and articular cartilage. Janus kinase/signal transducers and activators of transcription (JAK/STAT) signaling pathway is responsible for regulating the proliferation of fibroblast-like synoviocytes (FLS), synovitis, cartilage and bone destruction. NF-κB is an important transcription factor involved in inflammation that is activated by many cytokines such as TNF-α, RANKL, IL-1 and IL-6. Excessive activation of NF-κB can lead to abnormal apoptosis of FLS, stimulate the secretion of MMP, pro-inflammatory cytokines, inflammatory cells and mediators, and significantly promote bone destruction. FLS and chondrocytes with the function of secreting MMPs can degrade the components of joint extracellular matrix, leading to degeneration of joint cartilage. The activation of mammalian target of rapamycin (mTOR) pathway in synovial cells of RA patients promotes the proliferation of IL-17-induced FLS and the generation of osteoclasts. Wnt signaling pathway may be activated during the occurrence and development of RA, which controls the development of bone and the transformation of cartilage and bone, maintaining the dynamic balance of bone metabolism. Thereby, cytokines secretion, osteoclasts activation, and proline-matrix metalloprotein up-regulation contribute to bone erosion by activated Wnt signaling pathway. The toll-like family of receptors (TLRs) consists of myeloid differentiation factor (MyD88) dependent and independent pathways. The former leads to the production of pro-inflammatory cytokines, while the latter is related to the upregulation of IFN and major histocompatibility complex-Ⅱ. Both of which cause the production of inflammatory cytokines by NF-κB, and thus enhance the inflammatory cascade reaction of RA joints. Mitogen-activated protein kinases are important signaling pathways linking various cell surface proteins and inflammatory genes of the TNF family, chemokines, cytokines, and TLRs. The activation of p38, extracellular signal-regulated kinase (ERK) and c-Jun N-terminal kinase (JNK) in the families can regulate the expression of inflammatory factors, promote synovial inflammatory response, and mediate bone and cartilage destruction. In addition, it can activate macrophages and FLS, and promote the expression of inflammatory cytokines. As an important molecular mechanism of RA bone resorption and joint destruction, RANKL induces osteoclast differentiation by activating RANK receptor on the surface of osteoclast precursor. Osteoprotegerin is a natural bait of RANKL and inhibits osteoclast differentiation and bone absorption by preventing the binding of RANKL to RANK. In addition to autoinflammatory immunity, RA is associated with genetics, such as HLA-DRB1, CCR6, and CCR5 gene loci mutations, as well as environmental factors such as smoking (Ishigaki et al., 2017). And again, RA can be triggered by periodontitis, intestinal, oral and pulmonary microorganisms, and viral infections (Zhang et al., 2015; Du Teil Espina et al., 2019). With the development of molecular biology, numerous emerging RA biomarkers have been gradually elucidated. Correspondingly, there will be more novel and effective targeted therapies (Isaacs and Iqbal, 2019).

### Understanding of Pathogenesis of RA in Different Ethnic Medical Theories

Bi disease (痹病), first documented in the section of *Su Wen (Plain Conversation)·Bi theory of Huangdi Neijing*, is caused by the interaction between different forms of exopathogens (wind, cold, dampness and heat) and internal pathogens (phlegm and blood stasis). TCM classifies RA as the scope of “Bi disease”. Ancient and modern Chinese medicine classics called RA Li Jie (历节), Bai Hu disease, gout, Hexi Wind (鹤膝风), Wet Bi, Wan Bi, Bone Bi and Wang (尫) Bi (Zheng et al., 2017; Liu and Jiang, 2020). Modern and contemporary TCM masters declare that the pathogenesis of RA is essential empty and out solid (本虚标实), or asthenia of healthy Qi and sthenia of pathogenic factors (正虚邪实) (Bu et al., 2019). Specifically, liver and kidney, spleen and stomach, and Yang Qi deficiency, or Yingwei disharmony led to deficiency of healthy Qi, which causes the external or internal evil to take advantage of the empty and retain tendons bones arthrosis to induce RA. The prolonged RA course thus causes physical weakness and damage to the five Zang-organs. Therefore, according to the characteristics of pathogenesis and the purpose of addressing both symptoms and root causes, the treatment of RA is mostly based on the principle of removing pathogenic factors and supporting the healthy. Specifically, symptoms were alleviated by removing the pathogenic factors of internal and external wind, cold, dampness, heat, phlegm and blood stasis, while RA can be fundamentally annihilated by strengthening the physiological functions of the liver, kidneys, lungs, spleen and stomach. In addition, heat and toxin in TCM is equivalent to inflammation in western medicine, which can induce Bi, stasis and pain in RA patients. Therefore, heat-clearing and detoxicating therapy is used throughout the entire course of RA (Wang and Ma, 2018).

RA is called “Zhen Bu disease” (真布病) in Tibetan medicine, which also belongs to the category of Bi disease, divided into six categories: Rou Bi, Bone Bi, Mai Bi, Tendon Bi, White Bi and Black Bi. The understanding of RA by Tibetan doctors can be traced back to the *Four-Volume Medical Code* written by the famous Tibetan medical expert Yutuo·Yundan Gongbu in the 8th century AD, and it is believed that the intolerance of Qi and Blood and the increase of yellow water caused by the disorder of Long (隆), Chi Ba (赤巴) and Pei Gen (培根), induce RA (Huang et al., 2021). Based on the theory of Tibetan medicine, idiomatical external treatment of RA in the *Four-Volume Medical Code* includes Tibetan fire moxibustion and Tibetan medicine bath. Fire moxibustion is to use the heat of moxa cone to apply drugs to the joints of patients, so as to achieve the purpose of treating RA. The type and dosage of medicinal herb in Tibetan medicine bath can be adjusted according to the degree of joint pain, spasm, swelling, and blocked joint flexion and extension. It should be noted that the temperature of the Tibetan medicine extract with liquor should be controlled at 39–42°C for 30 min sitz bath each time, and sheltered from wind for about 2 h after the bath (Wang et al., 2017). Mongolian medicine classified RA as “Tulai disease” (图赉), which is caused by the imbalance of the three roots (Heyi-赫依, Xila-希拉, and Bada Gan-巴达干), as well Qisu (琪素), Yellow water (黄水) and Xila Wusu (希拉乌素). According to the clinical symptoms, RA is divided into the following three types: “Hari Tulai” (哈日图赉). “Zhagan Tulai” (查干图赉). “Alaga Tulai” (阿拉嘎图赉). RA is caused by excessive intake of spicy and stimulating food, excessive daytime sleep and fatigue, little exercise after satiation, strenuous activities, or invaded by cold wind and damp, and working in cold water for a long time. The main pathogenesis is that increased Xila Wusu, and Heyi as well Qisu fight each other for dominance, which aggress the small joints of hands and feet, the large joints of the whole body and the surrounding tendons. For a long time, the dysfunction of white vein, bone Heyi and Qisu in the affected area resulted in joint deformity, often involving the skin and viscera (Wu, et al., 2018; Guo, et al., 2021). Other ethnic medicine also has a unique understanding of RA. Dai medicine regards RA as Long-Meng-Sha-Throat disease (拢蒙沙喉). Affected by the person’s constitution and seasonal environment, the discordance of the Four Towers and Five Yun in the body leads to external evil (wind, hot, cold and damp evil) shuttling back and forth through joints, causing local Four-tower abnormality of the joint and inducing RA. Therefore, the focus of Dai medical treatment for RA is to remove the excessive external pathogenic factors in the local joints of the body, and restore the Four-tower state of the joint lesions, so as to improve the symptoms of RA (Pan et al., 2019). In the theoretical system of Zhuang medicine, RA is known as Gun Ke (滚克) and Fa Wang (发旺), which is mainly due to the patient’s physical weakness makes avails the evil poison (wind, wet, cold and hot poison) of the opportunity to get in and block the three channels (grain, gas and water channel) and the two roads (dragon and fire road), thus obstructing the functions of viscera, Qi, blood, and bone and flesh to cause RA (Zhong et al., 2018). RA is recorded in Tujia medicine as Zhongjie Feng (肿节风), which was caused by dampness together with wind and cold invasion of Three Yuan bores (三元孔窍). Its characteristic of the traditional external treatment to drive oil and fire is that the mixture of heated tung oil, ginger and shallot is used to thermally stimulate RA points. Moreover, supplemented by ironing, wiping, kneading and pressing to relax the muscles and stimulate the blood circulation, thereby ameliorating blood circulation and peripheral tissue nutrition for anti-inflammatory and detumescence (Ye et al., 2013; Nan et al., 2019). In addition, indoor buried in heated sand therapy also has a good effect on RA, known as “Wajol Mupasil” in Uygur medicine (Zhou et al., 2017).

To sum up, different ethnic medicine has unique theoretical understanding of RA, which is gradually formed into a relatively complete theoretical system in long-term clinical practice with a long history (Supplementary Figures S2 and S3). In addition to internal and external drugs, the emphasis on the wholeness and unity of the body enables the treatment strategies to engulf unique ethnic therapy, and take into account the importance of dietetic regulation, environment and patients’ psychology to RA disease with less adverse reactions. Therefore, we should recognize the irreplaceable advantages of different ethnic medicine in the treatment of RA and other chronic and stubborn diseases. In order to promote and develop distinctive ethnic therapy, it should be applied in clinical practice without hesitation. The updating of multiple disease surveillance methods has promoted the continuous in-depth understanding of RA, and the key diagnosis and treatment strategies have brought clinical benefits to more patients with RA. Accordingly, the integration of multi-center data, the introduction of AI-based deep learning and cloud computing are forward-looking and wise choice. Although it is just emerging, it is of great practical significance to guide the clinical treatment and basic research of RA.

### Status of Diagnosis and Treatment of RA

#### Clinical Diagnosis of RA

**FIGURE 4:**
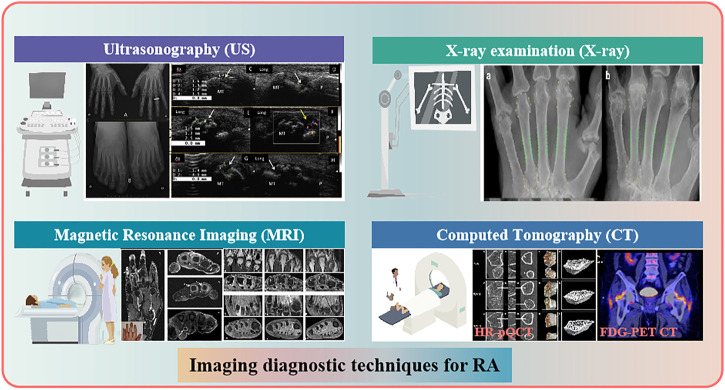
Different imaging diagnostic techniques for RA.

Currently, the clinical diagnosis of RA still lacks the generally accepted gold standard. In addition to skilled rheumatologists making decisions about RA based on typical clinical signs and symptoms, it is also necessary to selectively detect various serological indicators related to RA, such as RF, C-reactive protein, erythrocyte sedimentation rate, glucose-6-phosphate isomerase, IL-6, IL-1, anti-keratin, anti-perinuclear factor, anti-cyclic citrullinated peptide, anti-RA33, anti-Sa, and anti-p68 antibodies (Kumar et al., 2016; Malmström et al., 2017). However, the inevitable reality is that although the detection of serum RF is simple, rapid and high sensitivity, RF is also highly expressed in the serum of patients with systemic lupus erythematosus, Sjogren’s syndrome, hepatitis, tuberculosis and bacterial endocarditis, so there is no specific serological RA detection index at present. We therefore propose the introduction of multiple visual joint imaging at different stages of the pathogenesis of RA, such as ultrasonography (US), magnetic resonance imaging (MRI), X-ray plain film, computed tomography (CT), fluorescence optical imaging, and isotope radiography, which can more accurately track any further spread of the disease and develop a more rational therapeutic regimen (Schäfer et al., 2013; D’agostino et al., 2016; Kumar et al., 2016; Allen et al., 2018; Panayides et al., 2020) (Figure 4).

The US, including gray-scale ultrasonography, high-frequency color doppler ultrasonography, power doppler ultrasonography, and superb microvascular imaging, mainly reflects the early synovial thickness, articular cavity effusion, cartilage and blood flow signal strength in RA patients. On a two-dimensional scale, US can intuitively locate the eroded cartilage tissue in RA patients (Haavardsholm et al., 2016; Roux et al., 2019). MRI can visualize the joint structure and histopathological changes of healthy persons and RA patients with multiple plane images, including articular cavity effusion, synovial hyperplasia, marrow edema, articular cartilage and bone destruction, ligament, tendon and inflammatory exudation, especially the imaging of pannus. MRI is considered as the gold standard for clinical diagnosis of patients with early RA and plays an important role in follow-up, prognosis and efficacy evaluation (Baker et al., 2017; Hunt et al., 2017). X-ray examination of the bones and joints in RA patients, including normal X-ray plain film, digital tomosynthesis and digital X-ray radiogrammetry, the main objective is to evaluate joint space stenosis and bone erosion (Figueiredo et al., 2018). Three-dimensional reconstructed technology of CT can clearly display diverse angles of the articular surface and detailed bone. Radionuclide imaging is not a routine examination of RA and is just used for the differential diagnosis of difficult miscellaneous diseases and the exclusion of malignant tumors, but it shows certain advantages in some aspects. in recent years, some researchers have proposed that single photon emission computed tomography, based on the specific radioactive concentration patterns of each substance, can be used for the detection of angiogenesis in early RA with high sensitivity and specificity (Kubota et al., 2017). To the best of our knowledge, some near infrared fluorescence probes developed and synthesized in recent years have shown promising prospects for application *in vivo* RA animal models. An investigation on RA mice have shown that hypochlorous acid produced during the pathogenesis of RA can significantly enhance the fluorescence activity of the probe by breaking the C=N bond of the fluorescence probe, which may become a potentially sensitive monitoring method for the detection and evaluation of therapeutic effect and disease progression of RA (Feng et al., 2018). A novel near-infrared (NIR)-II fluorescent probe CH1055-WL may perform well in the detection of articular cartilage degeneration in early RA patients (Yi et al., 2019). IRDye800CW (a safe, and either oral or subcutaneously injectable NIR-II fluorescence agent) has been shown to be able to detect the process of collagen-induced arthritis in RA mice *in vivo* and in real time (Bhatnagar et al., 2019). All the above methods for clinical or preclinical RA examination have their unique advantages. However, the high cost and complex procedures are unacceptable to most rheumatologists and patients.

In summary, before conventional or emerging medical technologies can be approved for clinical diagnosis of RA disease activity, we need to conduct a cohort of clinical randomized trials to evaluate the clinical safety, effectiveness, accuracy, and range of application of each technique. In addition, and more importantly, reasonable statistical models for quantifying various RA images also play an important role in the identification of RA condition, prediction of progress trend and evaluation of therapeutic efficacy (Antill et al., 2016; Brown et al., 2017). Although the above imaging technologies have their own focus and advantages, available and valuable diagnostic information from images requires an experienced rheumatologist with time-consuming and laborious, and somewhat subjectivity. Advanced algorithms-trained DL or AI has attracted the attention of more clinical rheumatologists in image processing, information acquisition, diagnosis and treatment of various diseases including RA with the merit of efficient, reasonable, accurate and comprehensive halo.

### Western Medicine and Traditional Medicine for Treatment of RA

At present, most western drugs for RA are mainly anti-inflammatory, analgesic, immunosuppressive and hormone therapy, which aim to relieve patients’ discomfort symptoms and control the development of the disease, protect the function of the affected joints to the greatest extent, and relieve joint deformity. The relevant drugs available are categorized into nonsteroidal anti-inflammatory drugs (NSAIDs), disease-modifying anti-rheumatic drugs (DMARDs) and glucocorticoids (GCs). NSAIDs are primarily used to improve RA patients with severe joint pain and stiffness. But NSAIDs has relatively serious and high incidence of adverse reactions in the digestive system, mainly caused by COX-1 such as aspirin and indomethacin. DMARDs can prevent and delay the destruction of the articular bone structure of RA, including conventional synthetic DMARDs (cs-DMARDs) such as methotrexate (MTX), leflunomide, and sulphasalazine, as well as novel DMARDs (biologic DMARDs (b-DMARDs) and targeted synthetic DMARDs (ts-DMARDs) (Hazlewood et al., 2016). More and more clinical data have implied that novel DMARDs, including biologic DMARDs (b-DMARDs) and targeted synthetic DMARDs (ts-DMARDs), bring a new era to the treatment of RA. GCs is the most effective anti-inflammatory drug at present. The complex binding GCs with the corresponding receptors translocate into nucleus, reducing the transcriptional regulatory activity of NF-κB, and thus reducing the production of pro-inflammatory factors. Therefore, it produces powerful anti-inflammatory and analgesic effects and enables patients to rapidly relieve symptoms. However, it is well known that GCs does not prevent the progressive deterioration of RA and joint destruction. In contrast, GCs can cause severe infections, hormonal osteoporosis, aseptic osteonecrosis, and iatrogenic adrenocortical dysfunction or hyperactivity (Katz-Talmor et al., 2018). Given the risk of fractures, the researchers suggested that the addition of statins and TNF-α inhibitors, and the limited use of opioid, GCs and psychotropic drugs may reduce the risk of vertebral fractures in RA patients with cardiovascular disease and other joint fractures of RA patients (Ozen et al., 2019). Furthermore, although most rheumatologists prefer bDMARDs and GCs to control the inflammatory response of RA, they increase the risk of double infection after joint replacement or during routine treatment compared to csDMARDs and other RA drugs (George et al., 2019; Mahase, 2019). In conclusion, we suggest that rheumatologists should provide patients with a rational western drug treatment regimen for RA according to the latest official guidelines.

Every kind of ethnic medicine has taken their place in the era of contention of a hundred schools of thought. Although the medical theories of various ethnic groups are obscure and difficult to understand, there is no shortage of effective drugs and prescriptions for some intractable and chronic diseases, which exact effects have been widely recognized internationally. Ethnic drug interventions are inexpensive and generally affordable for patients in remission of RA. It can not only avoid the liver and kidney toxicity caused by western medicine, but also adjust the body immunity and improve the physique, so it is worth further study. However, when choosing the treatment regimen, rheumatologists should inquire the medical history of RA patients, and introduce related ethnic drugs under the guidance of ethnic medicine theory on the basis of existing western medicine treatment, or substitute ethnic drugs for western drugs with potential or serious adverse reactions. Secondly, the patient’s physique, applicable population, indications and contraindications of each drug should be mastered to achieve rational drug use. Supplementary Table S1 lists the representative ethnic drugs for RA.

Clinical investigation has confirmed that the efficacy of *Tripterygium wilfordii* Hook F (Lv et al., 2015) or tripterygium glycosides (TGs) tablets (a commercially available Chinese patent medicine containing TGs extract) (Wang et al., 2018) for RA is similar to that of MTX, and is better when combined with MTX. A clinical parallel, randomized and non-blind controlled efficacy evaluation trial suggested that MTX combined with sinomenine (SIN) may be an alternative to MTX with leflumide (Huang et al., 2019). Although existing a concomitant histamine-releasing anaphylaxis of SIN, its robust anti-inflammatory effects make it a good candidate for clinical treatment of RA (Huang et al., 2017). The n-butanol extraction of *Panax notoginseng* (Chang et al., 2007), and sodium tanshinone IIA (TIIA) sulfonate (Wang et al., 2019c) can play the role of anti-TNF-α and various inflammatory cytokines by inhibiting NF-κB and MAPK signaling pathways, improving the symptoms of the experimental RA models *in vitro* and *vivo*. *Paeonia lactiflora* Pallas and total glucosides of paeony (TGP) exert anti-inflammatory, analgesic, inhibiting synovial hyperplasia and angiogenesis, and therefore can be clinically applied in the treatment of RA (Zhang and Dai, 2012). Recent studies have shown that 12 weeks of TGP administration improves the symptoms of joint injury in RA rats by balancing intestinal microbes (Peng et al., 2019). Surprisingly, long-term clinical evaluation has showed that additional TGP supplementation reduced hepatic toxicity in RA patients treated with MTX and leflumide (Xiang et al., 2015). Quercetin, another anti-RA monomer, has been shown to act on anti-inflammatory, analgesic and anti-oxidant effects via the suppression of NF-κB and the activation of nuclear factor erythroid 2-related factor (Nrf2)/heme oxygenase (HO-1) pathway (Guazelli et al., 2018; Yang et al., 2018). *In vivo* and *in vitro* evidence also suggests that quercetin could maintain the Th17/Treg ratio, repress the activation of NLRP3 inflammasome (Yang et al., 2018), and downregulate the levels of inflammatory cytokines such as TNF-α, IL-1β, IL-6, and IL-17, MMP and monocyte chemoattractant protein (MCP-1) in experimental RA models (Haleagrahara et al., 2018). As a splendid herbal source of immune modulators, *Panax ginseng* and/or ginsenosides can improve the symptoms of RA by anti-TNF-α and anti-oxidant (Lee JI. et al., 2019b; Yi., 2019), and there were almost no obvious clinical adverse reactions in RA patients (Cho et al., 2018). In ayurvedic medicine, a crude extract mixture of *Azadirachta indica-*Nimbidin, mainly composed of nimbin, nimbinin, nimbidinin, nimbolide and nimbidic acid, has potential anti-inflammatory effects by inhibiting the functions of macrophages and neutrophils (Kaur et al., 2004), which partly explains the mechanisms of *Azadirachta indica* for treating RA in the folk for a long time. *Curcuma Longa* and its main ingredient curcumin are also a traditional herbal treatment for RA in India (Yang et al., 2019). Many evidence has confirmed that curcumin could inhibit RA-evoked inflammatory process, oxidative stress events (Manca et al., 2019), and synovial hyperplasia (Dai et al., 2018), by the inhibition of NF-κB signal transduction and induction of macrophage apoptosis (Wang et al., 2019a), as well targeting mTOR pathway (Dai et al., 2018). In addition, the inhibition of the production of inflammatory cytokines and the increased antioxidant defense capacity reported by *Swertia chirayita* (Lad and Bhatnagar, 2016) and *Nyctanthes arbortristis* (Rathore et al., 2007) also doomed its potential in the treatment of RA.

In summary, most ethnic herbal medicines are being valued as treatments or alternative combinations for RA. The gradual elucidation of the mechanisms of each drug and the positive clinical results have also subtly increased people’s recognition and acceptance of ethnic drugs. Somewhat imperfectly, due to the concept of different national medical culture and the traditional standards of drug utilization, the ethnic medicine inevitably has a gap with the understanding of diseases and the development of drugs in the modern medical system. However, the national medical law, which has been preserved for generations along the long evolution of human beings, is undoubtedly the precious property of all nations over the world. We should firmly believe that the preferential support of policies and the introduction of the emerging of modern advanced technology, such as multiple omics auxiliary disease biomarkers and drug targets screening, optimization of the extraction and separation technology, as well as the conspicuous advantages of explosive applications of AI in the medical field in recent years, the future will bring forth more ethnic medicine therapies to make up for the numerous helpless situations of western medicine in treating RA.

### Non-Drug Therapy for RA

Considering that the above-described western or ethnic drugs can induce more or less reversible or irreversible adverse reactions, many rheumatologists and patients are gradually turning to non-drug therapies for RA treatment. Among them, targeted-acupoint-strategy (TAS) can strengthen the body resistance to eliminate pathogenic factors by stimulating the acupoints in various parts of the body to dredge meridians, harmonize Yin and Yang. Currently, TAS clinical application includes electroacupuncture, moxibustion, gold thread acupuncture (Joo and Park, 2017). The effectiveness of plastering, liniment, and drug injection on acupoint, as well other physical manipulation of acupoints (such as acupoint laser irradiation, pressure, and blockade) has also been demonstrated. Similar therapies include fire needle, burning needle and picking needle therapy inherited and innovated by Zhuang and Yao nationality, as well as cautery and moxibustion therapy by Hui nationality (Qiao et al., 2011). It is worth noting that the thread moxibustion therapy of Zhuang medicine (Huang, 1986) and sand therapy of Uygur medicine (Niyazi, 2002) have been proved to have a good therapeutic effect on RA for a long time, and they were listed as the third and fourth representative items of national intangible cultural heritage by China intangible cultural heritage protection center in 2011 and 2014 respectively. Another interesting example is that the use of live bees to sting specific acupoints or lesions of RA patients, as well as the injection of bee venom (Son et al., 2007), which is also widely spread in Chinese folk and has a good effect on RA. Furthermore, scraping, wax therapy, Tai Chi, Five Animals Play, aerobic exercise, resistance training, and joint gymnastics also have a definite effect on RA.

Different medical systems have also focused on the role of diet in regulating RA. Low salt intake, moderate alcohol consumption, high quality balanced diet rather than a single diet, dietary fruits administration or short-term fasting may reduce the intensity of the autoimmune response by reducing the intake of dietary antigens (Basu et al., 2018). What’s more, reducing the intake of N-glycolyl-neuraminic acid and increasing the intake of unsaturated fatty acids, polyphenols, glycosides and flavonoids can reduce the inflammatory response and improve clinical symptoms of RA. Moreover, there are effective therapeutic strategies such as ozone self-blood therapy and personalized gene targeting therapy (Adkar et al., 2017), stem cell therapy, mineral spring bath, infrared and ultraviolet therapy. Compared with conventional therapies that target inflammatory cytokine biologics, more promising may be the technologies with different sources of membrane-coated drugs, such as neutrophil-coated nanoparticles, have a broad spectrum of anti-inflammatory effects for RA without identifying which inflammatory cytokines are involved and largely depending on the function of the candidate cells (Zhang et al., 2018). Therefore, the clinical efficacy of non-drug therapy as a supplement to drug therapy in treating RA should not be ignored. However, most of which lack the support of solid evidence-based medical evidence, making it difficult to provide molecular explanations for their efficacy from a scientific perspective. Besides, since non-drug therapy is largely dependent on the years of clinical experience of rheumatologists, we strongly recommend that RA patients choose an officially recognized institution for proper treatment. Furthermore, surgical treatment is an effective method once patients are ineffective in the later stages of medical treatment and accompanied by joint pain, deformity, limited movement and loss of function. Depending on the patient’s complaint, rheumatologists may choose synovectomy, joint fusion or artificial joint replacement (Maderbacher et al., 2018).

### Opportunities and Challenges of AI Integrated Medical Big Data in the Diagnosis and Treatment of RA

In view of a series of problems existing in RA diagnosis, identification, drug efficacy, prognosis and risk assessment, the application of AI involving multiple algorithms has shown irreplaceable advantages in many links of RA disease. Active shape models combined with X-ray images segmentation of the joint have perfectly realized the diagnosis and disease progression assessment of osteoarthritis (Seise et al., 2007). Fuzzy inference systems (Singh et al., 2012) or an artificial neural network (ANN) model of serum associated inflammatory factors training in patients with arthritis (Heard et al., 2014) can be used for early identification and diagnosis of arthritis. The scoring system involving ANN also improve the accuracy of arthritis risk prediction and assessment (Yoo et al., 2016). Considering the similarity of various types of arthritis, RA has a higher misdiagnosis rate. Fortunately, the image analysis based on 3D bone microstructure imaging combined with multiple intelligent algorithms can avoid RA misdiagnosis and with a higher precision of genetic algorithm (Akgundogdu et al., 2010). For articular cartilage injury that may occur in RA, MRI images segmentation based on voxel classification approaches may have potential in assessing articular cartilage damage (Shim et al., 2009). Further integration with ANN will help assess changes in cartilage volume during RA progresses (Hafezi-Nejad et al., 2017). By combining with other machine learning algorithms, such as support vector machine (SVM), random forest and naïve Bayes, it may be more helpful to obtain more information about cartilage damage parameters in RA patients (Du et al., 2018). Meanwhile, this computer-aided MRI image segmentation method can be used to evaluate wrist bone erosion and edema in RA patients (Crowley et al., 2011).

The first mention of AI in the diagnosis of RA, multi-center electronic health records should be attached importance to integrate the clinical characteristics of RA by using active or deep learning in machine learning-based electronic phenotype algorithms, providing valuable clinical references for the diagnosis and prognosis of RA (Norgeot et al., 2019). In the early 1990s, despite of few data sources and simple algorithms, scientists have tried to build a computer-aided medical decision-making systems for evaluation and management of RA patients through inductive learning (Kern et al., 1993). Considering the complex anatomy of human joints, the anatomic localization of individual joints is very important for orthopedic surgeons to diagnose digitized radiographs of RA. As joints space width (JSW) is narrowed by destroyed articular cartilage, an artificial neural network-based algorithm was firstly developed to determine the anatomical landmarks and JSW in joints of the hand for quantitative arthritis assessment instead of semi-quantitative scoring systems of radiography (Duryea et al., 2002). Its sensitivity to progressive RA assessment remains to be proven, although it is fully automated and has very little user interaction. Better still, the position and erosive contours of RA bone radiograph are determined by active shape models of computer aided algorithms (Langs et al., 2009). And it turns out that active appearance models (AAM) can be used to calculate the modeled shape and texture variation of RA associated joints. However, it is a sterner challenge for AAM to analyze medical images of contour disorder. While, robust AAM method can process gross disturbed images of RA patient’s joints based on gray-value appearance to some extent, but it cannot process the seriously disturbed joints (Beichel et al., 2005). In order to screen out novel and sensitive image-based biomarkers of RA, rigid and nonrigid image registration and analysis algorithms were employed to automatically quantify changes of ankle joints in RA rats by serial magnetic resonance imaging (Leung et al., 2006). Instead of subjective evaluation of RA progression based on visual inspection, the histopathological changes were thus assessed in a three-dimensional perspective, which will contribute to the development of RA candidate therapeutic agents. Similarly, the use of computer-aided diffuse optical tomography for feature extraction and image classification is helpful in the diagnosis of RA (Montejo et al., 2013a; b). Dynamika-RA software exclude the unrelated motion disturbance and other noise artefacts, shown in MRI images of RA patients, by using 2D and 3D registration algorithms, improving the sensibility to evaluate early efficacy and quantify synovial joint inflammation in RA patients based on automated voxel-by-voxel analysis algorithms (Kubassova et al., 2010). By introducing machine learning methods including kernel learning, neural network and linear discriminant algorithms, the electromyographic response (ER) acquisition from healthy and RA individuals during gait was recorded and analyzed (Nair et al., 2010). While measuring ER of RA patients during gait is of little significance for clinical diagnosis of RA, it can be used to partially assess the rehabilitative degree of RA patients after receiving any drug or non-drug therapy.

Secondly, AI also plays a strong function in exploring the complications and pathogenesis of RA. As one of the complications induced by RA, interstitial lung disease has been quantified by application of a computer-assisted MeVis PULMO 3D software (Marten et al., 2009). Although it saves time and has high reproducibility, due to fewer recruited volunteers, no pattern-based analysis of CT scan, signal attenuation of high cavity organs, such as respiratory tract and bronchus, and limited number of threshold value, therefore, longitudinal investigations and larger cohorts of RA patients with pulmonary disease are needed to further confirmed the effectiveness of this system, and reasonable algorithms should be added to enlarge the scope of the system. Hierarchical learning algorithm (MegaSNPHunter) was proposed to statistically identify and quantify the high-order local interactions of multiple single nucleotide polymorphisms (SNPs) in RA, which was simulated by Wellcome Trust Case Control Consortium. Shockingly, more interactive patterns among SNPs of RA were identified and quantified (Wan et al., 2009). In order to apply the results of whole genome data of RA to the disease diagnosis and related targeted drugs development, however, changes in global and false-positive interactions of RA-induced SNPs need to be further properly managed. In fact, the interaction between genes caused by RA at the chromosomal level that are far beyond the extent to which current technological means can reach. Therefore, it is advisable to apply the supervised machine learning including multi-step logical progressive algorithms and multiple statistical methods to the screening and verification of candidate target genes related to RA disease in the early stage of experiment or new drug development (Briggs et al., 2010). However, the complexity of RA-evoked genetic changes determines the complexity of algorithms and statistical methods, which should be kept in mind when we incipiently construct computer-aided algorithm models. Although the genetic variants will affect the phenotype of RA patients, it is not an exact match. Hence, the complete machine learning platform should include risk assessment and prediction of faulty interacting genes, so that preventive measures can be taken before risks occur (Kruppa et al., 2012). Amazingly, a divinable model of RA mortality and risk factors based on machine learning method-Random Survival Forests provides the possibility of risk aversion at the early stage of RA diagnosis (Lezcano-Valverde et al., 2017).

Third, AI is also attracting a lot of interest from pharmaceutical companies and pharmacologists around the world. TNF-α converting enzyme (TACE) inhibitors have been proven to have the potential to treat RA by inhibiting excessive TNF-α production. Cong *et al.* firstly develop four machine learning models, including SVM, *k*-nearest neighbor (*k*-NN), back-propagation neural network and C4.5 decision tree, for screening the inhibitory efficiency of various candidate compounds on TACE. After that, the authors evaluated the reliability of the established model by using two separate methods: 5-fold cross-validation and independent evaluation (Cong et al., 2009). We expect that the above updated multiple mechanical learning models can guide the discovery of more candidate compounds or pharmacodynamic groups acting on RA related biomarkers in the future, and provide an efficient and non-blind pre-screening for the development of RA treatment drugs. Recently, the combination of machine learning and glycomics has shown great potential for screening more available potential serum markers in RA patients (Chocholova et al., 2018), which will undoubtedly lead clinicians to a deeper understanding of RA. At the 2017 annual meeting of the American Association of Immunologists, twoXAR, Inc., an AI-driven biopharmaceutical company, announced that they have developed an integrative AI-driven bioinformatics drug discovery platform for rapid identification and validation of novel RA drug candidates, lead compounds, and a deeper understanding of RA pathophysiology, which could contribute to expand alternative treatment strategies for RA focusing on new targets.

**FIGURE 5:**
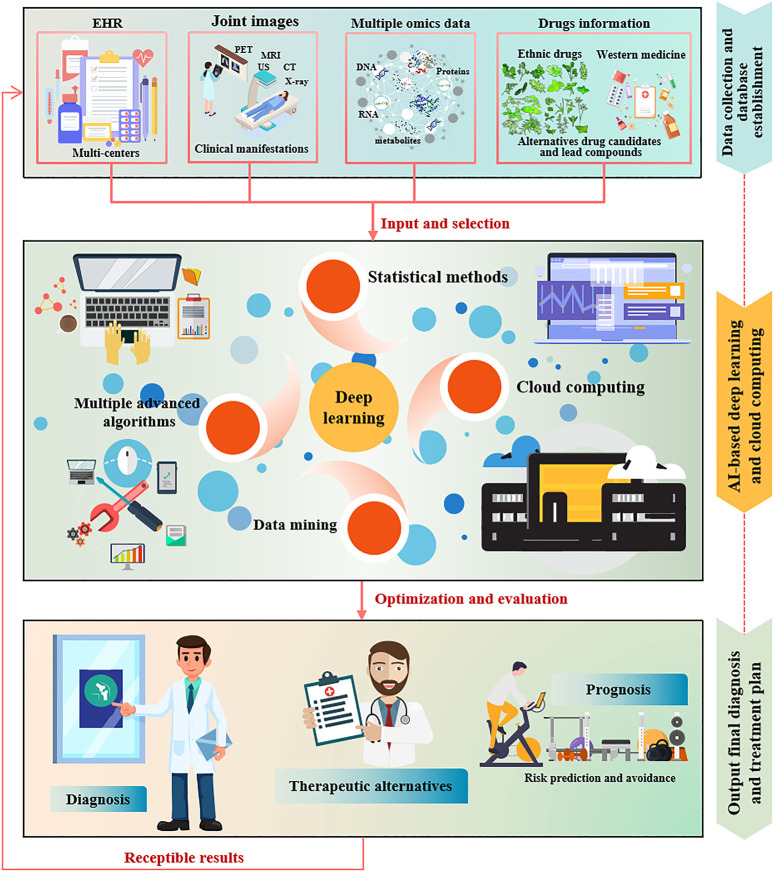
AI-assisted deep learning and cloud computing conceptual model for RA diagnosis and treatment.

Due to its powerful computing, analysis and induction functions, cloud computing attracts clinical and scientific researchers to deal with massive disease data obtained through proteomics, genomics, nucleic acid omics and intestinal microbial differences (Feng et al., 2017; Gibbs, 2017; Langmead and Nellore, 2018; Koppad et al., 2021). Although cloud computing is still in the conceptual attempt stage in the medical field, we have reason to believe that the application of cloud computing in RA will bring new opportunities for RA diagnosis, treatment, drug development and disease prognosis. In general, in addition to conventional RA diagnosis methods and new drugs development processes, data mining, machine learning and cloud computing are still in the early stages of RA diagnosis and prognosis, biomarkers and lead compounds screening. Figure 5 proposes a reasonable conceptual model of AI-assisted deep learning and cloud computing for RA diagnosis and treatment. Whereas, in the long run, these preclinical and clinical applications of computer-assisted methods will renovate current medical practice over the next two or 3 decades.

## Conclusions

AI has laid a scientific and technological foundation for the diagnosis and treatment system of RA in traditional medicine and western medicine, and the AI algorithm based on cloud data sharing is an indispensable part. Data acquisition and authenticity is our primary concern, these data mainly come from electronic medical record system of medical institutions, disease registration system, medical insurance system, public health survey and public health monitoring, birth/death registration database, genomics database, and other data generated in the process of disease prevention and health management (Wang et al., 2020; Zhu, 2020; Sun et al., 2021). Although integrated medical information, acquired by data mining, deep learning and cloud computing have been emerging in the diagnosis and treatment decision making of RA, there are still aspects worth further improvement and optimization. The first thing to point out is that reliable information on the etiology, dynamic course and prognosis, as well as multiple and effective treatment strategies of RA, requires the selfless dedication and close cooperation of clinical and scientific researchers worldwide. Primary clinical and scientific data on RA, including epidemiology, clinical and basic investigation, and traditional medical diagnostic methods, as well as potential targets and pharmacological mechanisms of various TCM/ethnic drug preparations or individual drugs should be selflessly uploaded and shared in the field in an officially authorized online database, so authenticity of the original data and timeliness of data sharing are critical. Secondly, reasonable and efficient computer algorithms, which contribute to the intelligent and efficient integration of all information related to RA disease should be updated technically in a timely manner. Interdisciplinary professionals in fields such as medicine and informatics or computer science, should be competent to leverage medical big data to facilitate RA. However, while narrowing the distance between patients with RA and therapists worldwide under the background of internet, it also increases the risk of patient privacy disclosure, which should not be ignored (Craig, 2016; Price and Cohen, 2019). In addition, we need to clearly recognize that AI can perform multifactor analysis of complex systems and find patterns in fragmented data to support processes such as disease diagnosis and drug discovery. However, AI cannot replace the diagnosis and treatment process of diseases, especially the spiritual comfort and deep communication for patients (Car et al., 2019). Not every RA patient can be accurately diagnosed and treated according to conventional medical means. To achieve excellent values for integrated medical big data of RA, therefore, reasonable protocols should be developed to determine when and where to use data mining, deep learning, and cloud computing to assist ethnic medicine in RA diagnosis and treatment. In summary, considering the conclusive defects drawn by the three above-mentioned methods in processes of data acquisition, processing and integration, the conclusions thus cannot effectively be used to guide and apply to clinical treatment of RA. We sincerely hope that in the era of medical big data, more detailed disease information for RA patients should be integrated together through reasonable of AI algorithms. Relatively novel RA diagnosis and treatment strategies thus could further guide different ethnic medicine around the world to better serve the clinical RA patients, and improve the quality of the survival of RA patients. But by comparison, diverse data sources, reasonable computer algorithms and machine simulation training models can make AI-assisted deep learning and cloud computing have considerable applications in RA diagnosis and treatment.
