# Supplementary material for: Practical Implementation of Artificial Intelligence-Based Deep Learning and Cloud Computing on the Application of Traditional Medicine and Western Medicine in the Diagnosis and Treatment of Rheumatoid Arthritis
Source: Front Pharmacol. 2021 Dec 23;12:765435. doi: 10.3389/fphar.2021.765435 (PMC8733656; doi:10.3389/fphar.2021.765435)
Supplement: Supplementary file 1 [file DataSheet1.docx]

**Supplementary materials**

**For**

**Practical Implementation of Artificial Intelligence-based Deep Learning and Cloud Computing on the Application of Traditional Medicine and Western Medicine in the Diagnosis and Treatment of Rheumatoid Arthritis**

*Shaohui Wang^1^*^†^*, Ya Hou^2^*^†^*, Xuanhao Li^3^, Xianli Meng^4^, Yi Zhang^1*^ and Xiaobo Wang^4*^*

^1^*School of Ethnic Medicine, Chengdu University of Traditional Chinese Medicine, Chengdu, China; ^2^**School of Pharmacy, Chengdu University of Traditional Chinese Medicine, Chengdu, China; ^3^Chengdu Second Peoples Hospital, Chengdu, China; ^4^**State Key Laboratory of Southwestern Chinese Medicine Resources, Innovative Institute of Chinese Medicine and Pharmacy, Chengdu University of Traditional Chinese Medicine, Chengdu, China*

^†^*These authors contributed to this work equally.*

***Corresponding Author:** Xiaobo Wang, E-mail: VitaDrwang@cdutcm.edu.cn. Yi Zhang, E-mail: zhangyi@cdutcm.edu.cn.

**Figure Legend for Supplementary Data**

**Supplementary Figure 1.** Multisystem diseases associated with RA. BCC, basal cell carcinoma; SCC, squamous cell carcinoma; CMD, coronary microvascular dysfunction; COPD, chronic obstructive pulmonary disease; ILD, interstitial lung disease.

**Supplementary Figure 2.** Theoretical interpretation of RA by different ethnic medicine.

**Supplementary Figure 3.** Exotic and different ethnic treatments for RA.

**FIGURE S1**


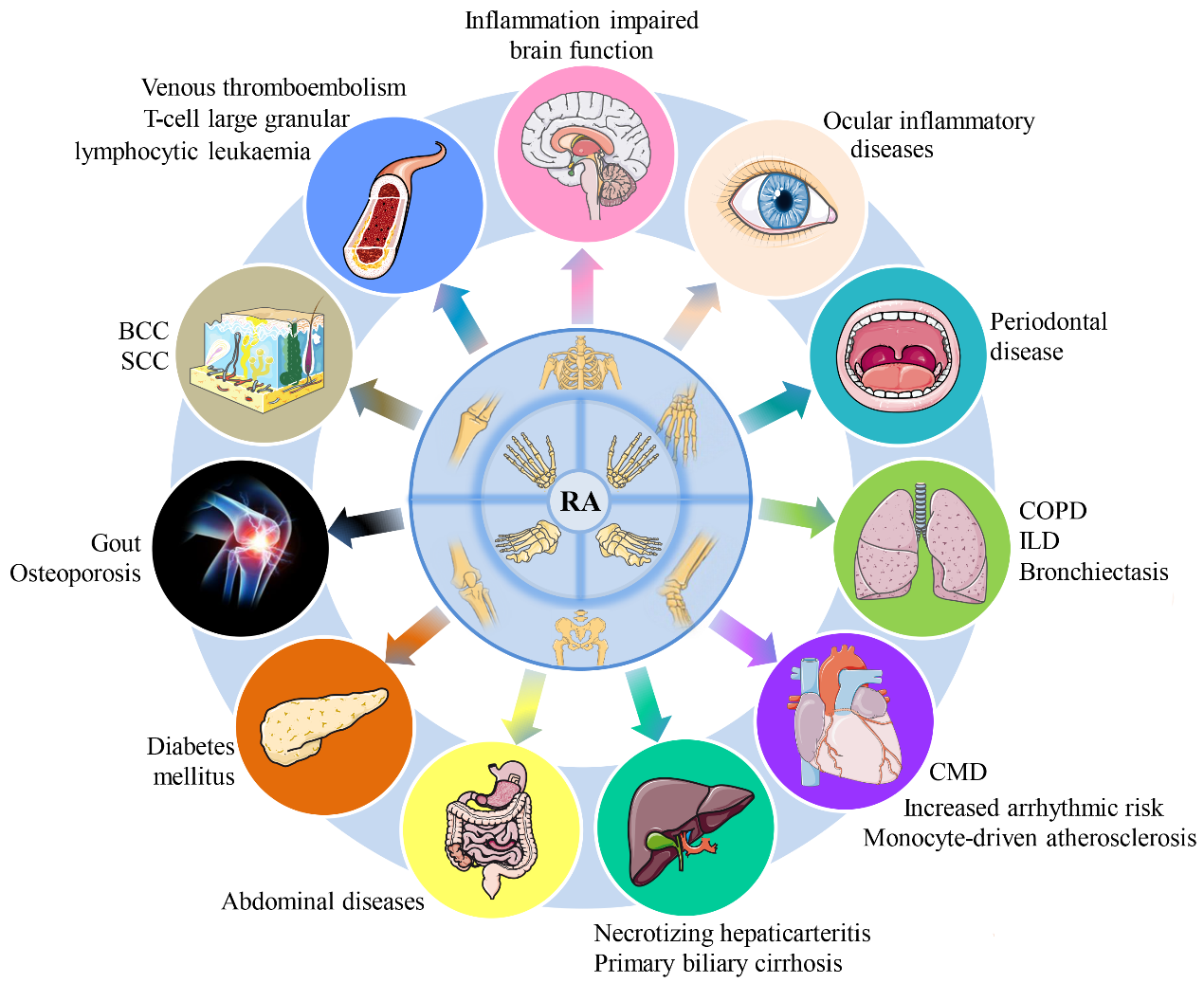


**FIGURE S2**

**
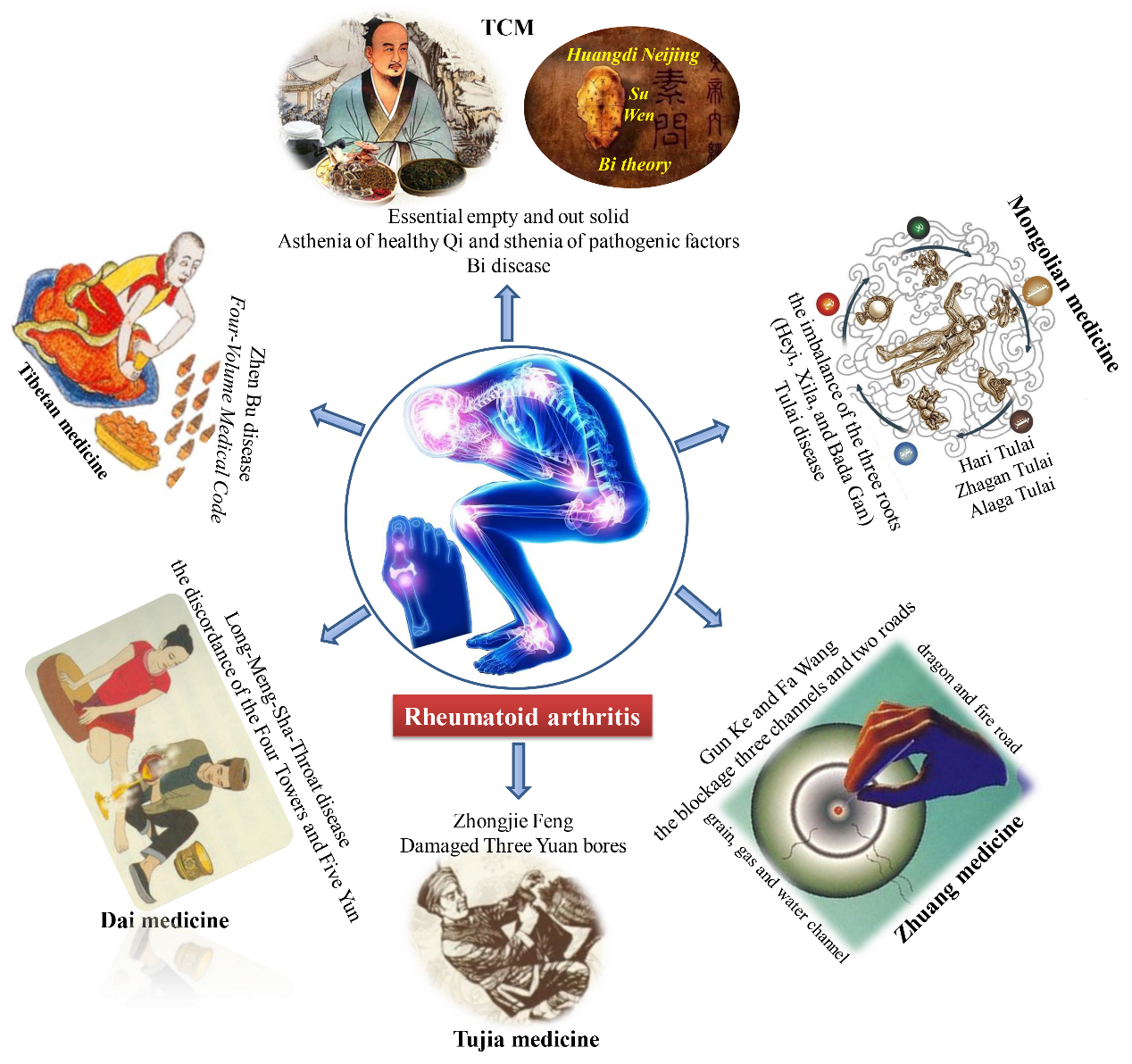
**

**FIGURE S3**

**
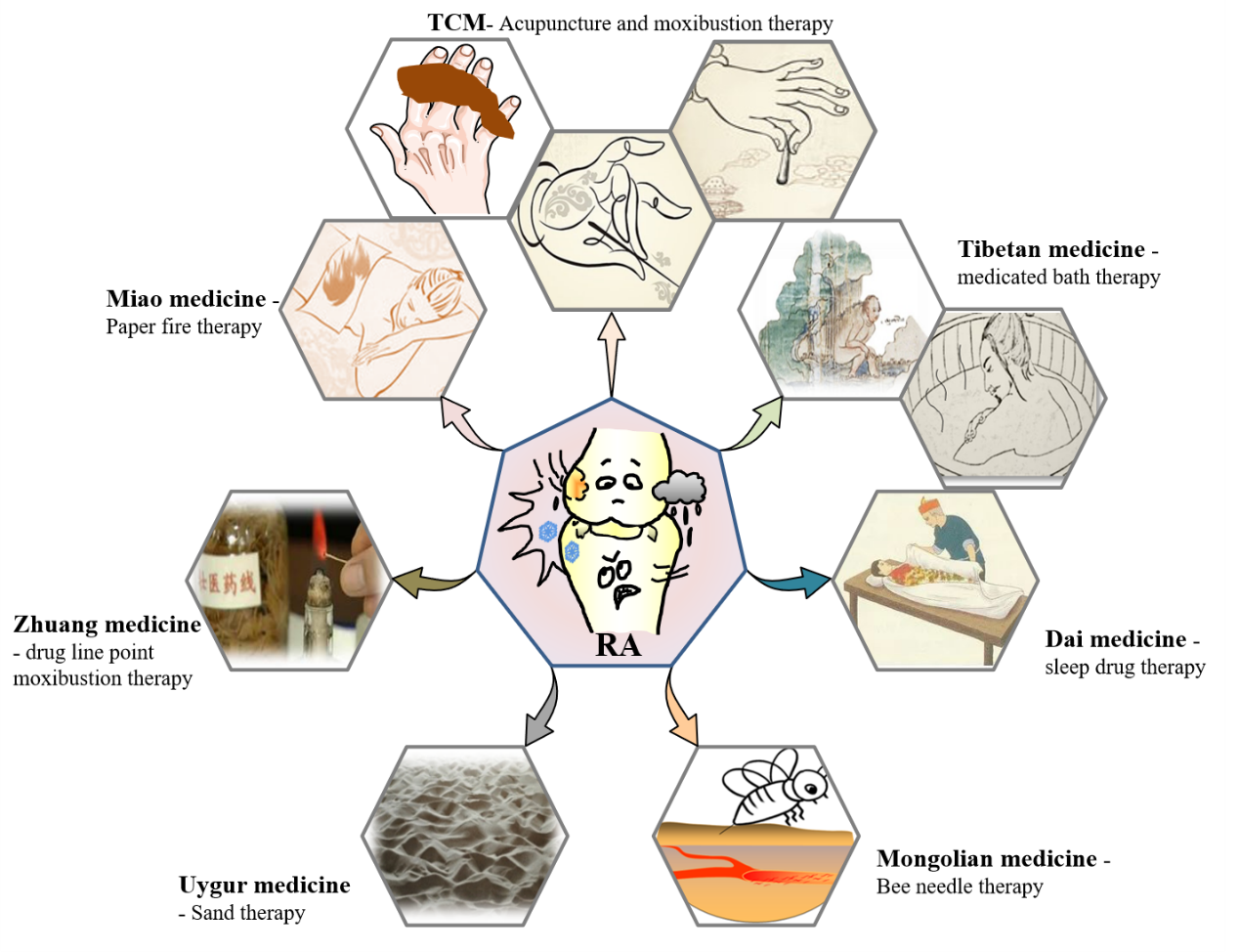
**

**Table Legend for Supplementary Data**

**Supplementary Table 1.** Representative herbal drugs and their medicinal parts for RA in various ethnic groups.

**Table S1**

| **Number** | **Chinese name of ethnic medicine** | **Latin scientific name of sources** | **Medicinal part** |
| --- | --- | --- | --- |
| **TCM** | | | |
| 1 | Lei gong teng | *Tripterygium wilfordii* Hook.f. | Roots, leaves and flowers |
| 2 | Qing feng teng | *Sinomenium acutum* (Thunb.) Rehd. et Wils. | Rattan stem |
| 3 | Ji xue teng | *Spatholobus suberectus* Dunn. | Rattan stem |
| 4 | Ding gong teng | *Erycibe obtusifolia* Benth. | Rattan stem |
| 5 | Wa e teng | *Tylophora ovata* (Lindl.) Hook. ex Steud. | Roots |
| 6 | Wei ling xian | *Clematis chinensis* Osbeck. | Roots and rhizomes |
| 7 | Fang feng | *Saposhnikovia divaricata* (Turcz.) Schischk. | Roots |
| 8 | Ren shen | *Panax ginseng* C. A. Mey. | Roots and rhizomes |
| 9 | San qi | *Panax notoginseng* (Burk.)F.H.Chen. | Roots and rhizomes |
| 10 | Shao yao | *Paeonia lactiflora* Pall. | Roots |
| 11 | Xi shu | *Camptotheca acuminata* Decne. | Fruits and roots |
| 12 | Wu tou | *Aconitum carmichaeli* Debeaux. | Roots |
| 13 | Qin jiao | *Gentiana macrophylla* Pall. | Roots |
| 14 | Deng zhan xi xin | *Erigeron breviscapus* (Vaniot) Hand.-Mazz. | Whole herb |
| 15 | Shi song | *Lycopodium japonicum* Thunb. | Whole herb |
| 16 | Du huo | *Heracleum hemsleyanum* Diels. | Roots |
| 17 | Yin yang huo | *Epimedium brevicornu* Maxim. | leaves |
| 18 | Tu fu ling | *Smilax glabra* Roxb. | Rhizomes |
| 19 | Dan shen | *Salvia miltiorrhiza* Bunge. | Roots and rhizomes |
| 20 | Fu pen zi | *Rubus chingii* Hu. | fruits |
| 21 | Bai hua she she cao | *Hedyotis diffusa* Willd. | Whole herb |
| 22 | Da huang | *Rheum officinale* Baill. | Roots and rhizomes |
| 23 | Huang qin | *Scutellaria baicalensis* Georgi. | Roots |
| 24 | Huang lian | *Coptis chinensis* Franch. | Rhizomes |
| 25 | Ma qian zi | *Strychnos nux-vomica* Linn. | Seeds |
| 26 | Ku dou zi | *Sophora alopecuroides* L. | Seeds |
| 27 | Chuan xiong | *Ligusticum chuanxiong* Hort. | Rhizomes |
| 28 | Li lu | *Veratrum nigrum* L. | Roots and rhizomes |
| 29 | Wu bei zi | *Rhus chinensis* Mill. | Cecidum |
| 30 | Dang gui | *Angelica sinensis* (Oliv.) Diels. | Roots |
| **Tibetan medicine** | | | |
| 31 | Yi shou cao | *Pterocephalus hookeri* (C. B. Clarke) Hock. | Whole herb |
| 32 | Shui bai zhi | *Myricaria gerynanica*(L.)Desv. | Twigs |
| 33 | Xi zang mao ru | *Rhamnella gilgitica* Mansf. et Melch. | Dry xylem |
| 34 | Du yi wei | *Lamiophlomis rotate* (Benth. ex Hook. f.) Kudo. | Above-ground part |
| 35 | Shui mu xue tu zi | *Saussurea medusa* Maxim. | Whole herb |
| 36 | Ta huang | *Rheum nobile* Hook. f. et Thoms. | Rhizomes |
| 37 | Lv xue | *Equus asinus* Linnaeus. | The blood of a horse donkey |
| 38 | Kuan jin teng | *Tinospora sinensis* (Lour.) Merr. | Vine stem |
| 39 | Ci bai | *Juniperus formosana* Hayata. | Roots |
| 40 | Da hua hong jing tian | *Rhodiola crenulata*(Hook. f. et Thoms.)H. Ohba. | Roots and rhizomes |
| **Mongolian medicine** | | | |
| 41 | Du song | *Juniperus rigida* S. et Z. | Branches, leaves and cones |
| 42 | Shou shen | *Gymnadenia conopsea* (L.) R. Br. | Tubers |
| 43 | Yan bai cai | *Bergenia purpurascens* (Hook. f. et Thoms.) Engl. | Rhizomes |
| 44 | Lan ci tou | *Echinops sphaerocephalus* L. | Roots |
| 45 | She xiang | *Moschus moschiferus* Linnaeus. | Dry secretions from mature male sachets |
| **Dai medicine** | | | |
| 46 | Ya zui hua | *Justicia adhatoda* L*.* (Adhatoda vasica Nees). | Whole herb |
| 47 | Bi ba | *Piper longum* L*.* | Fruit cluster |
| 48 | Da ye qian jin ba | *Curculigo capitulate* (Lour.) O. Kuntze. | Roots |
| 49 | Da ye xian mao | *Curculigo capitulate* (Lour.) O. Kuntze. | Roots and rhizomes |
| 50 | He huan | *Albizia julibrissin* Durazz. | Bark, inflorescence or bud |
| 51 | Tie geng hai tang | *Chaenomeles speciose* (Sweet) Nakai. | Fruits |
| 52 | Da bo gu dan | *Gendarussa ventricosa*(Wall.)Nees | Young shoots with leaves |
| 53 | Da ye gou teng | *Uncaria macrophylla* Wall. | Stem branches with hooks |
| 54 | La chang shu | *Cassia fistula* Linn. | Roots, bark, fruit pulp and seeds |
| 55 | Mai ma teng | *Gnetum montanum* Markgr. | Vines, roots and leaves |
| 56 | Wen shu lan | *Crinum asiaticum* L. var. *sinicum* (Roxb. ex Herb.) Baker. | Leaves or bulbs |
| 57 | Qing niu dan | *Tinospora crispa* (L.) Hook. f. et Thoms. | Roots |
| **Uygur medicine** | | | |
| 58 | Xin jiang jia long dan | *Gentianella turkestanorum* (Gand.) Holub | Whole herb |
| 59 | Chui guo teng | *Capparis spinosa* L. | Roots and leaves |
| **Hui medicine** | | | |
| 60 | Tie bang chui | *Aconitum pendulum* Busch. | Roots |
| 61 | Hai tong pi | *Aralia chinensis* L. | Bark |
| 62 | Tang gu te rui xiang | *Daphne tangutica* Maxim. | Stem and root bark |
| 63 | Chuan shan long | *Dioscorea nipponica* Makino. | Rhizomes |
| 64 | Fu niu hua | *Damnacanthus indicus* Gaertn. | Flowers |
| 65 | Man tuo luo | *Datura stramonium* Linn. | Leaves, flowers, seeds |
| 66 | A wei | *Ferula sinkiangensis* K. M. Shen. | Plant resin |
| 67 | Cang er | *Xanthium sibiricum* Patr. | Ripe fruit with involucre |
| 68 | Lao guan cao | *Geranium wilfordii* Maxim. | Above-ground part |
| 69 | Mu jin ji er | *Caragana frutex (L.)* C. Koch. | Flowers |
| 70 | Ye xi gua miao | *Hibiscus trionum* Linn. | Roots or whole herb |
| 71 | Hua bei bai qian | *Cynanchum hancockianum* (Maxim.) Al. Iljinski. | Whole herb |
| 72 | Tou gu cao | *Speranskia tuberculata* (Bunge) Baill. | Whole herb |
| 73 | Hui xiang | *Foeniculum vulgare* Mill. | Fruits |
| 74 | Luo tuo peng zi | *Peganum harmala* L. | Seeds |
| 75 | Mu bie zi | *Momordica cochinchinensis* (Lour.) Spreng. | Seeds |
| 76 | Ai na xiang | *Blumea balsamifera* (L.) DC. | Leaves and twigs |
| 77 | Ya xiang mao | *Cymbopogon nardus* (L.) Rendle. | Whole plant |
| 78 | Qian ma | *Urtica fissa* E. Pritz. | Whole herb |
| 79 | Hai shi hua | *Costazia costazii* Audouin. | The dried skeleton of an animal liverwort or liverwort |
| 80 | Xue lian hua | *Saussurea involucrata* (Kar. et Kir.) Sch.-Bip. | Whole plant with flowers |
| 81 | Niu xi | *Achyranthes bidentata* Blume. | Roots |
| 82 | Huang hua wu tou | *Aconitum coreanum* (Levl.) Rapaics | Roots |
| 83 | Shui long gu | *Polypodiode snipponica*(Mett.)Ching | Rhizomes |
| 84 | Jie gu mu | *Sambucus williamsii* Hance. | Stem branches |
| 85 | Qian jin teng | *Stephania japonica* (Thunb.) Miers. | Roots or stem leaf |
| 86 | Chai zi gu | *Luisia morsei* Rolfe. | Root or whole herb |
| 87 | Qie gen | *Solanum melongena* L. | Roots |
| 88 | Guai liu | *Tamarix chinensis* Lour. | The tender branches and leaves |
| 89 | Gou qi zi | *Lycium chinense* Mill. | Fruits |
| **Miao medicine** | | | |
| 90 | Hei gu teng | *Periploca forrestii* Schltr. | Roots |
| 91 | Ba jiao feng | *Alangium chinense* (Lour.) Harms. | Roots or leaves, flowers |
| 92 | Jian xue fei | *Caesalpinia cucullata* Roxb. | Roots or root bark |
| 93 | Jin mao gou ji | *Cibotium barometz* (L.) J. Sm. | Rhizomes |
| 94 | Zhui feng san | *Lysimachia paridiformis* Franch. var. *stenophylla* Franch. | Whole herb or Roots |
| **A-Chang medicine** | | | |
| 95 | Zhu sha gen | *Ardisia crenata* Sims. | Roots |
| **Bai medicine** | | | |
| 96 | Nuo mi tuan | *Gonostegia hirta* (Bl.) Miq. (*Memorialis hirta* (Bl.) Wedd.) | Whole herb |
| **Buyi medicine** | | | |
| 97 | Da xue teng | *Sargentodoxa cuneate* (Oliv.) Rehd. et Wils. | Rattan stem |
| 98 | Hong hua | *Carthamus tinctorius* L. | Flowers |
| **Dong medicine** | | | |
| 99 | Dian bai zhu | *Gaultheria yunnanensis* (Franch.) Rehd. | Whole herb |
| **Maonan medicine** | | | |
| 100 | Hong hua qing teng | *Illigera rhodantha* Hance. | Whole herb |
| 101 | Wu zhu yu | *Euodia rutaecarpa* (Juss.) Benth*. (Tetradium ruticarpum* (A. Juss.) T. G. Hartley) | Fruits |
| **Qiang medicine** | | | |
| 102 | Hong mao wu jia pi | *Acanthopanax giraldii* Harms. | Stem or root bark |
| 103 | Qiang huo | *Notopterygium incisum* C. T. Ting ex H. T. Chang. | Roots and rhizomes |
| **She medicine** | | | |
| 104 | Di nie | *Melastoma dodecandrum* Lour. | Whole herb |
| **Yao medicine** | | | |
| 105 | Kuo ye shi da gong lao | *Mahonia bealei* (Fort.) Carr. | Leaves |
| **Li medicine** | | | |
| 106 | Bai le | *Acanthopanax trifoliatus* (L.) Merr. | Root or root bark, young branches and leaves |
| 107 | Jie gu cao | *Sambucus chinensis* Lindl. | Whole herb |
| 108 | Dian ba jiao feng | *Alangium yunnanense* C. Y. Wu ex Fang. | Root, fibrous root, or root bark |
| **Ayurvedic medicine in India** | | | |
| 109 | Jiang huang | *Curcuma longa* L. | Rhizomes |
| 110 | Yin du zhang ya cai | *Swertia chirayita* (Roxb.ex Flemi)Karsten. | Whole herb |
| 111 | Yin du ku lian shu | *Azadirachta indica* A. Juss (Meliaceae). | Seeds, leaves and bark |
